# Lithium Prescription in Unipolar Depression: Findings from a Nationwide Greek Cohort Study

**DOI:** 10.1192/j.eurpsy.2026.10603

**Published:** 2026-07-31

**Authors:** V. Karageorgiou, I. Michopoulos, M. Hadjulis, P. Mitrou, K. Mathioudakis, P. Skapinakis, R. Gournellis

**Affiliations:** 1 National and Kapodistrian University of Athens; 2 Hellenic Ministry of Health; 3 IDIKA SA - e-Government Center for Social Security Services, Athens; 4 University of Ioannina, Ioannina, Greece

## Abstract

**Introduction:**

Treatment-resistant depression (TRD) is highly prevalent and can affect one in three patients with unipolar depressive disorders. Lithium is an established option in the treatment of TRD. However, real-world data on how this recommendation is perceived by prescribers, as well as psychopharmacological and diagnostic correlates, remain scarce.

**Objectives:**

To investigate the frequency, diagnostic predictors, and prescribing patterns of lithium in patients diagnosed with unipolar depressive disorders in Greece, with a focus on polypharmacy and potential drug interactions.

**Methods:**

We analysed a large Greek nationwide prescription database covering all ICD-10 unipolar depression codes for one calendar year. Logistic regression models were used to identify diagnostic predictors of lithium prescription, adjusting for age, sex, and psychiatrist involvement. We examined co-prescription patterns and potentially interacting drugs. Sensitivity analyses were conducted to exclude patients with coexisting psychiatric disorders during the observation period.

**Results:**

A total of 9,218,025 prescriptions were retrieved for 753,142 patients who received at least one unipolar depressive disorder diagnosis. Of these, 1,314 patients (0.174%) were prescribed lithium (people prescribed lithium, PPL). PPL were younger, with males overrepresented, and were more likely to have been treated by a psychiatrist. Disorder severity markers such as psychotic symptoms (adjusted odds ratio (aOR) 6.05 (5.21, 6.81)) and mixed presentations (aOR 7.12 (5.11, 9.08)) were predictive of lithium prescription. The most commonly prescribed primary agent was venlafaxine (38% of PPL), while atypical antipsychotics were the most frequently co-prescribed drug class (51%). The historically first described effective combination of lithium + tricyclic antidepressant was prescribed to 7% of PPL.

One in thirteen PPL received a potentially interacting drug, predominantly in the class of antihypertensives.

In sensitivity analyses, the proportion of PPL among those that consistently received only a unipolar depression was even lower (0.08%).

**Conclusions:**

Lithium prescription in unipolar depressive disorders remains unpopular and is predominantly given in those with severe depression. Newer antidepressants are favoured against the classical combinations of lithium and tricyclic antidepressants. Despite proven efficacy and a sustained positioning in the treatment algorithms for treatment-resistant depression, other alternatives are preferred.

**Disclosure of Interest:**

None Declared

